# Prevalence and Risk Factors of Metabolic-Associated Fatty Liver Disease in Children with Down Syndrome at a Tertiary Care Center

**DOI:** 10.3390/jcm14093239

**Published:** 2025-05-07

**Authors:** Maria D. Karaceper, Maria-Jose Villegas, Sanathan Sadh, Sierra Kawesa, Jamie Strain, Asha Nair, Alissa Dupuis, Mary Pothos, Ming-Hua Zheng, Mohit Kehar

**Affiliations:** 1 Division of Pediatric Medicine, Children’s Hospital of Eastern Ontario, University of Ottawa, Ottawa, ON K1H 8L1, Canada; mkaraceper@cheo.on.ca (M.D.K.); nair@cheo.on.ca (A.N.); adupuis@cheo.on.ca (A.D.); pothos@cheo.on.ca (M.P.); 2 Division of Pediatric Gastroenterology, Hepatology and Nutrition, Children’s Hospital of Eastern Ontario, Ottawa, ON K1J 9B7, Canada; mvillegas@cheo.on.ca (M.-J.V.); sanathansadh23@rcsi.ie (S.S.); sierrakawesa@gmail.com (S.K.); jstrain@cheo.on.ca (J.S.); 3 MAFLD Research Center, Department of Hepatology, The First Affiliated Hospital of Wenzhou Medical University, Wenzhou 325000, China; 4 Key Laboratory of Diagnosis and Treatment for the Development of Chronic Liver Disease in Zhejiang Province, Wenzhou 325000, China

**Keywords:** pediatric MASLD, metabolic dysfunction

## Abstract

**Background:** The global rise of metabolic-associated fatty liver disease (MAFLD) in children is particularly concerning in high-risk groups such as those with Down Syndrome (DS), who have an elevated risk of obesity and insulin resistance. Despite increasing recognition of MAFLD in pediatric populations, data on its prevalence and risk factors among children with DS in Canada remain limited. **Method:** This retrospective study reviewed medical records of children with DS at the CHEO Down Syndrome Clinic (2013–2023). A diagnosis of MAFLD required evidence of hepatic steatosis on imaging, lab markers, or biopsy, along with the presence of metabolic risk features. Demographic, laboratory, and diagnostic data were analyzed. **Results:** Among 503 children with DS (231 females, 271 males; median age: 172 months), 54 (10.7%) had MAFLD. The MAFLD group was older (median age: 205 vs. 163 months, *p* = 0.0002) and had higher BMI (31.39 vs. 20.5, *p* < 0.0001). Most cases (47/54) were diagnosed via ultrasound, and 49/54 met MAFLD criteria due to excessive adiposity. Lab results showed a median ALT of 35 U/L, triglycerides of 4.4 mmol/L, and LDL cholesterol of 2.59 mmol/L. FibroScan in 13 children revealed a median transient elastography of 5.3 kPa. BMI was the strongest predictor of MAFLD (OR: 1.2, 95% CI: 1.1–1.2). **Conclusions:** The DS clinic-based prevalence of MAFLD underscores the need for proactive screening and early intervention. BMI was the strongest predictor, emphasizing targeted management strategies. Further research is needed to refine diagnostic approaches and improve outcomes.

## 1. Introduction

Pediatric non-alcoholic fatty liver disease (NAFLD) is characterized by persistent hepatic steatosis in individuals under 18 years; it includes a broad clinical spectrum from simple steatosis to non-alcoholic steatohepatitis (NASH), a progressive and inflammatory form that can lead to fibrosis, cirrhosis, and liver failure [1,2]. It is now recognized as a leading cause of chronic liver disease worldwide in children with estimated prevalence of approximately 10%, but it can rise to between 30% and 70% among children with obesity, depending on the diagnostic criteria and study methodology [1,2]. Recent advancements have reframed this condition as metabolic dysfunction-associated fatty liver disease (MAFLD), emphasizing the central role of metabolic dysfunction, including obesity, insulin resistance, and dyslipidemia, in its pathophysiology [3,4]. More recently, the term metabolic dysfunction-associated steatotic liver disease (MASLD) was introduced, further underscoring the relationship between metabolic dysfunction and hepatic lipid accumulation [5]. Whether referred to as MAFLD or MASLD, both terms highlight the central role of metabolic dysfunction in the pathophysiology of this prevalent condition.

Down syndrome (DS), the most common chromosomal condition, is associated with a broad spectrum of comorbidities, including liver disease [6] and metabolic dysfunction. Children with DS are particularly vulnerable to MAFLD due to their unique metabolic profile, characterized by a significantly higher prevalence of obesity (23–70%), insulin resistance, and dyslipidemia, all key risk factors for hepatic steatosis [6,7]. This vulnerability is likely influenced by several factors, including a lower resting metabolic rate, reduced physical activity, a preference for energy-dense, nutrient-poor diets, and an elevated risk of conditions such as hypothyroidism and diabetes [8]. Evidence of hepatic steatosis in patients with DS was reported as early as 1967 [9], and available limited studies do share a strong link. An Italian cohort found MAFLD prevalence rates of 45% in non-obese and 82% in overweight/obese children with DS, compared to 5.7% and 33%, respectively, in the general European pediatric population [10]. This group also identified a genetic basis for the increased risk of MAFLD in children with DS. Specifically, the PNPLA3 rs738409 variant was associated with elevated interleukin (IL)-6 levels. PNPLA3 is one of the most commonly implicated genetic variants in MAFLD, highlighting its significant role in disease development across populations [11,12]. Metabolic dysfunction can be shaped by unique healthcare systems and geographic factors [13]; hence, country/geographic specific data are needed. To date MAFLD-related data in Canada in children with DS is absent. To address this knowledge gap, our study aimed to describe a cohort of patients diagnosed with MAFLD followed at the Children’s Hospital of Eastern Ontario (CHEO) DS clinic over the past decade. The DS Clinic at CHEO actively follows over 300 children, providing comprehensive care to children with DS from infancy to adolescence; this offers an opportunity to address this knowledge gap. The findings will not only provide data on clinic-based prevalence of MAFLD in children with DS in Canada but will also contribute to the advancing understanding of MAFLD in high-risk pediatric populations, laying the groundwork for future targeted interventions and improved long-term outcomes. This amplified risk underscores the need to better understand the burden of MAFLD in this high-risk group.

## 2. Methods

This retrospective observational study analyzed the medical records of children with DS who attended the DS Clinic at the CHEO between 1 January 2013, and 31 December 2023. Ethics approval was granted by the CHEO Research Ethics Board (REB #20240015). Pediatric patients under 18 years of age with a confirmed diagnosis of DS were eligible for inclusion. Exclusion criteria included incomplete medical records or insufficient follow-up data. The study strictly adhered to privacy regulations, with all data de-identified before analysis to ensure confidentiality and compliance with institutional policies.

Data Collection—Data were extracted from medical records by trained research personnel using standardized Case Report Forms within the secure RED Cap platform. Collected variables included demographic information such as age and sex, detailed clinical history, and relevant laboratory findings. Weight status was categorized using BMI-for-age cut points recognized by the World Health Organization and the Centre for Disease Control. These categories are defined as underweight ≤5th percentile, normal or healthy weight from the 5th percentile to <85th percentile, overweight ≥85th to <95th percentile, and obese as ≥95th percentile. Key laboratory parameters included liver enzymes (ALT, AST, and GGT), lipid profiles, glucose levels, and additional cardio-metabolic markers. Elevated age and sex-specific ALT levels (defined as >22 (U/L) for girls and >26 U/L for boys) were recorded. Abnormal lipid profiles were categorized based on established Canadian pediatric guidelines to classify lipid levels [14]. Homeostasis model assessment (HOMA) was calculated using fasting insulin (mU/mL) multiplied by fasting plasma glucose (mmol/L) divided by 22.5. Lower HOMA values indicate higher insulin sensitivity. HOMA-IR values were interpreted as indicative of insulin resistance when exceeding standard pediatric thresholds, typically ranging from 2.5–3.0 as normal [15]. Elevated HOMA-IR value (>3.16) was considered consistent with insulin resistance [16]. Imaging data, including ultrasound and transient elastography (FibroScan) results, were reviewed to assess liver steatosis and stiffness. Additional information on comorbidities was also recorded. MAFLD diagnosis was defined by the presence of hepatic steatosis confirmed through imaging, biomarkers (ALT), or histology, in addition to at least one of the following criteria: overweight or obesity, prediabetes or type 2 diabetes, or evidence of metabolic dysregulation. Metabolic dysfunction was defined by the presence of at least two risk factors, including increased waist circumference, hypertriglyceridemia, low HDL cholesterol levels, impaired fasting glucose, or a triglyceride-to-HDL cholesterol ratio greater than 2.25 [3].

Data Management—All collected data were entered into the RED Cap database by trained personnel. Data accuracy and completeness were ensured through regular audits conducted by the research team. Any discrepancies identified during these audits were resolved collaboratively. De-identified data were securely stored in accordance with CHEO’s institutional data protection guidelines.

Statistical Analysis—Descriptive statistics were employed to summarize demographic, clinical, and laboratory characteristics of the study population. Continuous variables, such as liver enzyme levels, were reported as medians with interquartile ranges or means with standard deviations, depending on their distribution. Categorical variables, including the presence of specific metabolic risk factors, were summarized as counts and proportions. Comparative analyses between groups were performed using t-tests or Mann–Whitney U tests for continuous variables and chi-square tests for categorical variables. Logistic regression was used to identify predictors of MAFLD and evaluate factors associated with its progression. *p* values < 0.05 were considered statistically significant and statistical analysis was performed by Stata 10.

## 3. Results

The study included 503 children with Down syndrome (DS) over a 10-year period, consisting of 231 females and 271 males (Table 1). The median age of the cohort was 172 months (approximately 14.3 years), with an interquartile range (IQR) of 100–212 months. The median weight was 44 kg, and the median BMI was 21.75. Among the cohort, 54 children (10.7%) were diagnosed with MAFLD representing the clinic-based prevalence over a 10-year period. This included 29 females and 25 males. Children with MAFLD were significantly older than those without the condition (median age 205 months vs. 163 months, *p* = 0.0002) and heavier with a median weight of 66 kg (*p* < 0.0001), and a median BMI of 31.39 (*p* < 0.0001), with a median BMI percentile of 98.

**Figure 1:**
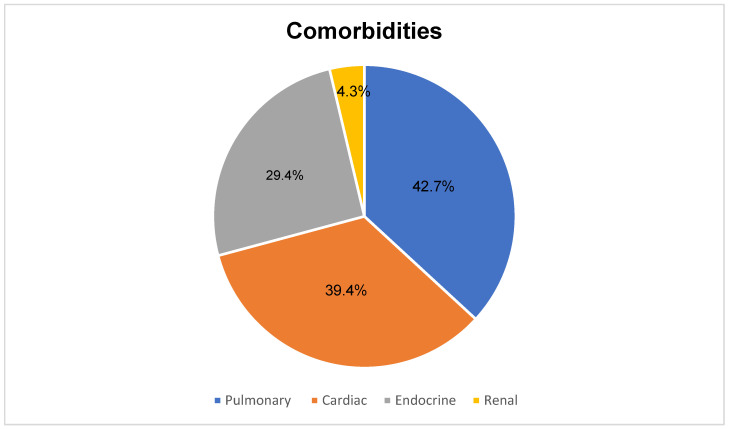
Comorbidities in the MAFLD cohort. Some children had more than one system involved, hence the % will be more than 100.

Within the MAFLD cohort children, four were within the 5th to <85th BMI percentile, six were between the 85th and 95th percentiles, and 44 were above the 95th percentile. The median systolic and diastolic blood pressures were 113 mmHg and 71 mmHg, respectively. Diagnosis was primarily based on imaging (48 out of 54 cases), with hepatic steatosis confirmed through ultrasound. Notably, 49 children met the diagnostic criteria due to evidence of excessive adiposity. In most cases (39/54, 72.2%), the diagnostic workup was initiated following significant weight gain. Symptoms at the time of diagnosis were relatively uncommon, with abdominal pain reported in 7.4% of cases and dyspepsia in 1.9%. Comorbidities were highly prevalent within the cohort, reflecting the broader medical complexities associated with DS (Figure 1).

### 3.1. Laboratory Findings

Laboratory evaluations revealed elevated markers of liver function and metabolic dysregulation (Table 2). Notably, 82.8% of females (24/29) had ALT levels > 22 U/L, while 68% of males (17/25) had ALT levels > 25 U/L. Lipid profile abnormalities were also observed, with elevated triglycerides, total cholesterol, HDL, and LDL. Specifically, 24.1% (13/54) had abnormal total cholesterol, 18.5% (10/54) had abnormal LDL, and 18.5% (10/54) had abnormal HDL levels. Elevated triglycerides were found in 3.7% of children under 9 years of age and 38.9% of children aged 10 or older, with seven children having an abnormal triglyceride-to-HDL ratio (>2.25). Metabolic markers showed a median fasting glucose of 5.15 mmol/L (IQR: 4.9–5.7), fasting insulin of 182 pmol/L (IQR: 134–410), and HbA1c of 5.3% (IQR: 5.1–5.5). The median HOMA-IR was 6.6, with 18.5% of children (10/54) exceeding the threshold of 3.16, indicating significant insulin resistance. Liver stiffness measurements using FibroScan in 13 children revealed a median value of 5.3 kPa, with a CAP score of 262 dB/ms. Liver biopsies were performed in two children, neither of whom showed evidence of cirrhosis.

### 3.2. Comparative Analysis and Predictors of MAFLD

Multivariate analysis (Table 3) identified BMI as a key predictor of MAFLD, with an odds ratio (OR) of 1.2 (95% CI: 1.1–1.2). This indicates that each unit increase in BMI is associated with a 20% increase in the odds of developing MAFLD in this population.

## 4. Discussion

This study highlights the significant burden of MAFLD in children with DS, an already vulnerable population due to their unique metabolic profiles. Among the cohort, 10.7% of children met the diagnostic criteria for MAFLD, with older age and higher BMI emerging as significant predictors. This study fills an important gap in the literature by providing much-needed data on MAFLD in Canadian children with DS and underscores the importance of early detection and intervention in this high-risk group. Future studies are needed to identify additional risk factors and improve diagnostic accuracy and outcomes for children with DS.

With the increased life expectancy of children with DS, a growing prevalence of non-communicable diseases has been observed. Metabolic syndrome and adiposity (obesity) are common in children with DS, significantly elevating their risk of complications associated with metabolic dysfunction [6,7]. Identifying the exact causes of increased obesity risk in DS remains challenging, but contributing factors may include reduced physical activity, unhealthy dietary habits, endocrine disorders, and abnormalities in adipokines, like leptin and adiponectin, commonly described in DS [8,17]. These dysfunctions, coupled with shared pathophysiological mechanisms, often lead to complications such as MAFLD. Additionally, children with DS exhibit higher oxidative stress [18], which may exacerbate hepatic inflammation in the context of MAFLD [19], highlighting the critical importance of early detection and management.

In our study, 10.7% of children with DS were diagnosed with MAFLD, notable given that the diagnosis was based on stringent criteria including imaging and metabolic dysfunction [3]. The findings are consistent with global trends, wherein children with DS exhibit disproportionately higher rates of obesity and insulin resistance, major contributors to MAFLD development. Importantly, 81.5% of children in the MAFLD group had BMI ≥95th percentile, reinforcing the central role of obesity as a modifiable risk factor. Among the full cohort, approximately 160 children had a BMI >95th percentile, with 54 meeting diagnostic criteria for MAFLD suggesting a rate of about 30% in the subgroup with obesity, as noticed earlier in studies [20]. Our findings also align with earlier research, such as an Italian cohort study that reported MAFLD in 64% of a selected subgroup of children with DS, based on extensive workup including adiponectin levels. However, selection bias was noted in that study, as it included only 84 children from a DS clinic population of 280. Interestingly, the same group also identified MAFLD in children with normal BMI supporting the need for broad screening. Other studies have similarly reported elevated ALT levels in children with DS and high BMI [21,22] indicating a potential risk of MAFLD in appropriate clinical settings.

Comorbidities were prevalent in our MASLD cohort, reflecting the complex medical needs of children with DS. Notably, cardiac conditions were present in 39.4% of children, while endocrine disorders, such as hypothyroidism and type 2 diabetes, affected 29.5%. Pulmonary conditions, including asthma, were also highly prevalent (42.7%). Comorbid conditions are well-documented in children with DS [23] and may significantly exacerbate the risk and progression of MAFLD. This highlights the needs for comprehensive, multidisciplinary management strategies [24] in children with DS.

The laboratory profiles of children with MAFLD in our study revealed significant metabolic dysregulation, characterized by elevated liver enzymes and lipid abnormalities. ALT levels were elevated in 82.8% of females and 68% of males, reflecting ongoing hepatic injury. Dyslipidemia as seen in other studies [21,25,26] was prevalent, with 38.9% of children aged 10 years or older showing elevated triglycerides, along with other lipid irregularities. Furthermore, 18.5% of children exhibited a triglyceride-to-HDL ratio > 2.25, a recognized marker of cardiometabolic risk [27] and hepatic steatosis [3]. This finding is particularly significant in the context of DS, where cardiac conditions (congenital heart diseases) are a leading cause of morbidity and mortality [28]. Similarly, cardiac events are a major cause of mortality in MAFLD [29], compounding the severity of this synergistic interplay between the two conditions. While abnormal lipid profiles may partly result from metabolic dysfunction, genetic factors such as the combined loss of Down syndrome critical region (DSCR-1) and apolipoprotein E are also implicated [30]. This dual loss has been shown to induce liver-specific metabolic abnormalities while paradoxically reducing atherosclerotic plaques, leading to elevated serum cholesterol levels and sporadic vasculopathy [30]. Additionally, insulin resistance emerged as a key feature in our cohort, with a median HOMA-IR of 6.6 and 18.5% of children showing HOMA-IR > 3.16. This underscores its critical role in driving both metabolic and hepatic complications in children with DS and highlights the importance of addressing these interconnected risk factors early in their clinical management.

In our study, BMI emerged as the strongest predictor of MAFLD, with an odds ratio of 1.2. This finding corroborates previous literature highlighting the critical role of adiposity in the pathogenesis of MAFLD [12]. This is also not surprising as 72.2% of children diagnosed with MAFLD underwent evaluation due to weight gain, and not often secondary to symptoms. Age also significantly influenced MAFLD risk, with older children (median age 205 months) more likely to develop the condition. This likely reflects the cumulative impact of prolonged metabolic dysfunction and underscores the need for early intervention to mitigate risk. Studies in animal models of Down syndrome have shown that aging exacerbates oxidative stress and liver fibrosis, supporting the importance of early detection [31]. Additionally, the BMI trajectory in children with DS shifts as early as 3 years in girls and 5 years in boys, with a rapid increase around 14 years. This pattern suggests that as children age, the risk of BMI-associated metabolic dysfunction, including MAFLD, rises, highlighting the importance of age-appropriate screening and interventions to address this escalating risk [32].

While weight management is a cornerstone of MAFLD prevention and treatment, Implementation can be particularly challenging in children with DS due to additional barriers such as developmental delays, feeding difficulties, and limited physical activity [33,34,35]. The lack of Canadian data on MAFLD in children with DS has historically impeded the development of effective management strategies. By providing valuable insights into the epidemiology and clinical characteristics of MAFLD in this population, our findings establish a foundation for future research and policy development. Geographic and healthcare system differences significantly influence metabolic dysfunction, underscoring the importance of tailoring interventions to the Canadian context. Other strengths of this study include comprehensive data collection and the use of standardized diagnostic criteria for MAFLD, enhancing the reliability of findings. However, our results must be interpreted within the context of limitations. The retrospective design may have introduced biases, particularly since not all children with obesity underwent comprehensive metabolic and hepatic evaluations due to the lack of standardized protocols, indicating that the true prevalence of the condition may be underreported in our study. Routine screening and awareness for MAFLD in children with obesity in Canada remains limited, as demonstrated by findings from a national study conducted by our group [36,37]. Furthermore, the small MAFLD cohort size and single-center design restrict the generalizability of our findings to other diverse geographic settings. Future research should focus on prospective studies to better understand the longitudinal progression of MAFLD and evaluate the effectiveness of targeted interventions. These efforts will be critical to improving long-term outcomes and addressing the unique challenges faced by children with DS.

## 5. Conclusions

In conclusion, this study highlights the high rates of MAFLD in Canadian children with DS, driven largely by excessive adiposity and compounded by a high burden of comorbidities. These findings emphasize the urgent need for early screening and comprehensive management strategies to address the unique metabolic challenges faced by this population. Further research is warranted to explore the long-term outcomes of MAFLD in DS and the effectiveness of tailored interventions.

## Figures and Tables

**Table 1:** Summarizing the baseline characteristics of the cohort.

Variable	Value
Number of participants	503
Sex (Female/Male) full cohort	231/271
Median age, months	172
Median weight, kg	44
Median BMI	21.75
Children with MAFLD (%)	54 (10.7%)
Sex (Female/Male) MAFLD cohort	29/25
Median age of MAFLD group, months	205
Median weight of MAFLD group, kg	66
Median BMI of MAFLD group	31.39
Median systolic BP, mmHg	113
Median diastolic BP, mmHg	71
MAFLD diagnosis based on imaging	48/54 (88.9%)
MAFLD diagnosis due to adiposity	49/54 (90.7%)

**Table 2:** Presents the laboratory markers and their corresponding median values along with the interquartile ranges (IQR) for various parameters related to liver function and metabolic regulation in the study population. ALT (Alanine Aminotransferase), AST (Aspartate Aminotransferase), GGT (Gamma-Glutamyl Transferase), ALP (Alkaline Phosphatase), INR (International Normalized Ratio), HbA1c (Glycated Hemoglobin), CAP (Controlled Attenuation Parameter), FibroScan (Transient Elastography), HDL (High-Density Lipoprotein), LDL (Low-Density Lipoprotein).

**Laboratory Marker**	**Median**	**IQR**
Total bilirubin	5 µmol/L	3–10 µmol/L
ALT	35 U/L	26–49 U/L
AST	26 U/L	22–38 U/L
GGT	30 U/L	23–50 U/L
ALP	315 U/L	107–231 U/L
Albumin	41 g/L	39–43 g/L
INR	1.04	1–1.9
Ferritin	82 µg/L	57–134 µg/L
Creatinine	64 µmol/L	52–80 µmol/L
Triglyceride	4.4 mmol/L	0.9–1.91 mmol/L
Total cholesterol	1.32 mmol/L	3.75–5.19 mmol/L
HDL cholesterol	1.1 mmol/L	1.01–1.25 mmol/L
LDL cholesterol	2.59 mmol/L	1.97–3.18 mmol/L
Fasting glucose	5.15 mmol/L	4.9–5.7 mmol/L
Fasting insulin	182 pmol/L	134–410 pmol/L
HbA1c	5.3	5.1–5.5
Liver stiffness (FibroScan, N-13)	5.3 kPa	4.7–6.6 kPa
CAP score	262 dB/ms	220–315 dB/ms

**Table 3:** Univariate and multivariate logistic regression analysis for predictors of MAFLD.

**Variable**	**Univariate Analysis**			**Multivariate Analysis**		
	**OR**	**95% CI**	***p*-Value**	**OR**	**95% CI**	***p*-Value**
Age (months)	1.008	1.003–1.012	<0.0001	0.997	0.99–1.003	0.4
Sex	0.707	0.40–1.20	0.23	–	–	–
(Female = Ref)						
BMI	1.19	1.14–1.25	<0.0001	1.2	1.10–1.20	<0.001

## Data Availability

The original contributions presented in this study are included in the article. Further inquiries can be directed to the corresponding author.

## Abbreviation

ALT (Alanine Aminotransferase), AST (Aspartate Aminotransferase), BMI (Body Mass Index), CAP (Controlled Attenuation Parameter), CHEO (Children’s Hospital of Eastern Ontario), DS (Down Syndrome), GGT (Gamma-Glutamyl Transferase), HbA1c (Hemoglobin A1c), HOMA-IR (Homeostasis Model Assessment of Insulin Resistance), IQR (Interquartile Range), LDL (Low-Density Lipoprotein), MAFLD (Metabolic Dysfunction-Associated Fatty Liver Disease), MASLD (Metabolic Dysfunction-Associated Steatotic Liver Disease), NAFLD (Non-Alcoholic Fatty Liver Disease), PNPLA3 (Patatin-like Phospholipase Domain-Containing Protein 3), TG (Triglycerides), HDL (High-Density Lipoprotein), REB (Research Ethics Board), U/L (Units per liter), and OR (Odds Ratio).
